# Simple equations for complex physiology: can we use VCO2 for calculating energy expenditure?

**DOI:** 10.1186/s13054-016-1251-3

**Published:** 2016-03-21

**Authors:** Pierre Singer

**Affiliations:** Critical Care Department, Institute for Nutrition Research, Rabin Medical Center, Beilinson Hospital, Petah Tikva, 49100 Israel; Sackler School of Medicine, Tel Aviv University, Tel Aviv, Israel

In a recent article, Stapel et al. [1] gave a practical and easy solution for the evaluation of energy expenditure in critically ill patients. Predictive equations are routinely used to determine energy needs and to guide the prescription of calories in critically ill patients [2]. However, their accuracy is very poor, resulting in both over- and underfeeding [3], and thus confounding the validity of many studies based on these equations. Indirect calorimetry remains the gold standard to determine calorie requirements, as well as to calculate energy expenditure (EE) from oxygen consumption (VO2), carbon dioxide production (VCO2) and nitrogen excretion (NM) measurements [4]. These devices are still seldom used since they may be expensive, require expertise and have several technical limitations [5]. Moreover, the most accurate device, the Deltatrac II (GE, Finland) is not widely available and other new devices have still to achieve its accuracy [6].

From the basic equation:

$$ \mathrm{E}\mathrm{E}\ \left(\mathrm{kcal}\right) = 3.581\ \mathrm{V}\mathrm{O}2\ \left(\mathrm{L}\right) + 1.448\ \mathrm{V}\mathrm{C}\mathrm{O}2\ \left(\mathrm{L}\right)-1.773\ \mathrm{urinary}\ \mathrm{nitrogen}\ \left(\mathrm{g}\right) $$

it is clear that the most important measurement for EE is VO2. A 10 % error in VO2 causes a 7 % error in EE while a 10 % error in VCO2 causes a 3 % error in EE [7]. Most devices are measuring VO2 and VCO2 or VO2 alone. EE derived from VCO2 alone was suggested already 25 years ago [8], using estimates of the energy equivalents of CO_2_ (energy expended/CO_2_ produced; EeqCO2). Taking into account the variations in CO_2_ related to starvation or artificial enteral feeding, tracer techniques demonstrated that the calculation of resting energy expenditure (REE) from CO_2_ production should not employ a universal value for VCO2. Nevertheless, measurement of VCO2 and the replacement of VO2 by VCO2/0.84 have been proposed to calculate EE [8]. This general value of 0.84 is the result of the arithmetic mean of the respiratory quotient (RQ) of the three main macronutrients: (1 + 0.809 + 0.707)/3 = 0.84. Since the measurement of VCO2 is available in many ventilator devices, it was suggested that this may be an easy and inexpensive way to calculate EE. Mehta et al. [9] used VCO2 alone in critically ill children to calculate EE and suggested that the REE may be obtained by measuring VCO2 through integrated devices in the ventilator or by a stand-alone monitor. The modified Weir equation (REE, kcal/day = 5.5 × VCO2 (L/min) × 1440 using a fixed RQ of 0.89) was compared with predictive equations and found to be much more accurate [7]. However, there was an inherent inaccuracy due to the fixed RQ. When a RQ macro (based on the ratio of carbohydrate to fat in the diet) was used based on the ratio between carbohydrates to fat in the diet, a closer agreement was obtained between measured and REE derived from VCO2 alone, reaching a mean bias for agreement between measured REE and VCO2-derived REE of −2.0 %, but with wide limits.

Sandra Stapel and colleagues [1] have proposed an even more sophisticated approach to achieve better accuracy using VCO2 alone in ventilated critically ill patients, extracting RQ from the nutrition regimen for each evaluation and not using a fixed value. When comparing this approach to mean 24 hour indirect calorimetry-based EE, bias was shown to be low, i.e., 141 ± 153 kcal/day and 7.7 % of the gold standard. In addition, it was more precise than the more frequently used equations (limits of agreement −166 to +447 kcal/day). These results may encourage physicians who do not have access to direct calorimetry but have ventilators equipped with VCO2 measurement modified by an adapted RQ, to derive EE from this measurement in order to more appropriately target the calorie prescription.

However, while the use of complicated mathematics resulted in good precision and low bias, the concept does not reflect the complexity of physiology in the critically ill patient. First the administered nutrients are only partially absorbed in these patients. Thus, small intestine glucose absorption is markedly impaired, independently of duodeno-cecal transit time [10] while lipid absorption is reduced by almost half when compared with healthy volunteers [11]. Secondly, endogenous glucose production is not depressed despite nutrition administration; adding a load of endogenous carbohydrates to the nutrient administration and autophagy provides endogenous lipids, carbohydrates and protein [12]. Third, secondary to stress, there is significant insulin resistance as well as obligatory lipolysis [13] and severe proteolysis which nutrients are unable to inhibit [14]. Finally, body substrate oxidation obtained by indirect calorimetry is far from the nutrient administration [15], making the correlation between the prescription and the respiratory quotient more difficult.

## Conclusion

Stapel et al. based their theory on the fact that the absorbed macronutrients determine the RQ, arguing that what is administered is utilized. While this approach may be preferred to predictive equations, it cannot reflect the complex physiologic changes seen in critically ill patients. Inaccuracies inherent in these types of calculations or measurements may explain why some interventional nutrition studies fail to achieve positive clinical outcomes.

## Abbreviations

EE: energy expenditure
NM: nitrogen excretion
REE: resting energy expenditure
RQ: respiratory quotient
VCO2: carbon dioxide production
VO2: oxygen consumption
